# Temporal Trends in the Investigation, Management and Outcomes of Acute Appendicitis over 15 Years in the North of England: A Retrospective Cohort Study

**DOI:** 10.1007/s00268-022-06586-x

**Published:** 2022-05-18

**Authors:** Jared Bhaskar, Ross C. McLean, Keir Bhaskar, Leo R. Brown

**Affiliations:** 1 St George’s University of London, Cranmer Terrace, Tooting, London, SW17 0RE England, UK; 2 Queen Elizabeth Hospital Site, Queen Elizabeth Avenue, Gateshead, NE9 6SX England, UK; 3 Department of Medicine, Imperial College London, Exhibition Road, South Kensington, London, SW7 2BX England, UK; 4 Royal Infirmary of Edinburgh, Little France, Edinburgh, EH2 2EQ Scotland, UK

## Abstract

**Background:**

Acute appendicitis is a common surgical emergency with an estimated lifetime prevalence of 8.6% for males and 6.7% for females. Despite the frequency of presentation, considerable variation in clinical practice exists. Our study aimed to explore temporal trends in the investigation, treatment and outcomes for patients with appendicitis between 2002 and 2016.

**Methods:**

Data collected included all patients aged ≥16 years across the NHS trusts in Northern England between 01/01/2002 and 31/12/2016 diagnosed with appendicitis. Patient demographics, co-morbidity and management strategies were included. Outcomes of interest were length of stay and inpatient mortality.

**Results:**

Over a 15 years period, 22,137 patients were admitted with acute appendicitis. A consistent male preponderance (n = 11,952, 54%) was observed, and median age increased over time (2002–2006: 36.4 vs. 2012–2016: 39.5, *p* < 0.001). Comorbidity of patients also increased (*p < *0.001) in recent years. Computed tomography (CT) use increased from 0.8 to 21.9% (*p < *0.001) over the study period. Following CT scanning, there was a longer time to theatre (1.22 vs. 0.70 days, *p < *0.001), and patients were more frequently managed non-operatively (23.8% vs. 5.7%, *p < *0.001). The utilisation of laparoscopic approaches significantly increased from 4.1 to 70.4% (*p < *0.001). Laparoscopic patients had a shorter median length of stay (2.97 days) when compared with open surgery (4.44 days) or non-operative (6.19 days) patients. The 30-day mortality rate was 0.33% overall and decreased with time (*p* = 0.004).

**Conclusions:**

CT and laparoscopic surgery are increasingly utilised in the management of appendicitis. Along with other advances in clinical practice, they have led to reduced lengths of stay and mortality.

## Introduction

Appendicitis is a common pathology worldwide that requires urgent treatment [1]. The UK incidence is approximately 81 cases per 100,000 person-years [2] and rose during the twentieth century. There is a lifetime prevalence of 8.6% for males and 6.7% for females [3]. Despite the lower prevalence, a higher rate of negative appendicectomy has been observed in females [4]. This is likely reflective of a greater difficulty in establishing a diagnosis where ovarian pathologies can present similarly to appendicitis [4, 5]. The overall negative appendicectomy rate in the UK of 20% is comparatively higher than other European countries (6%) [4].

The role of computed tomography (CT) in the diagnostic pathway for appendicitis has been debated. Potential benefits include improved diagnostic accuracy, identification of complicated pathology and of patients who may benefit from conservative treatment [6]. Disadvantages include requiring contrast to improve diagnostic accuracy, radiation exposure and increased cost, when compared to abdominal ultrasound [6]. The sensitivity and specificity of CT are estimated to be 95 and 94%, respectively, providing high yield diagnostic value, with even low-dose CT achieving equivalent accuracy [6]. Ultrasound (US) can be used in isolation or sequentially with CT as part of the diagnostic work up [6]. While US is free from ionising radiation exposure and useful for identifying gynaecological pathology, the appendix can be difficult to visualise and sensitivity and specificity for appendicitis are poor [7].

The World Society of Emergency Surgery (WSES) and European Association of Emergency Society (EAES) have proposed guidelines [8, 9] recommending routine risk scoring based on clinical assessment and biochemistry alongside imaging. These guidelines suggest the use of laparoscopic surgery in all cases of appendicitis, unless contraindicated [8, 9]. Laparoscopic appendicectomy carries significant advantages over open for reducing pain intensity on postoperative day one, wound infections, length of hospital stay and time to resume normal activities [10, 11]. The use of antibiotics to treat uncomplicated appendicitis has also been explored. Studies have not demonstrated a sound conclusion in efficacy of antibiotics compared to the standard appendicectomy [12, 13] but have highlighted the high risk of recurrence [14].

The aim of this study was to evaluate trends in clinical presentation, changes in the use of CT, management approach and outcomes for patients diagnosed with appendicitis across NHS hospitals in the North of England over a 15 years period.

## Methods

Data for hospital admissions are routinely collected by the Health and Social Care Information Centre to provide Hospital Episode Statistics (HES) [15]. Following Caldicott approval, data from acute NHS foundation trusts in the North of England were retrieved (“Appendix 1”). All emergency admissions under a general surgeon between 1 December 2001 and 30 November 2016 were requested. The data fields requested are listed in (“Appendix 2”)*.* Patient data were irreversibly anonymised prior to being sent to the authors.

### Data definitions and management

The cohort comprised of patients aged 16 years and older, admitted under a general surgeon as an emergency with a diagnosis of appendicitis. Postal codes were converted to Index of Multiple Deprivation (IMD) scores using the online postcode conversion tool [16] and then converted to deprivation quintiles [17]. Weekends were defined as Saturday and Sunday, including bank holidays, and weekdays defined as Monday to Friday. Data on co-morbidities were generated by mapping secondary ICD-10 diagnostic codes to the relevant co-morbidity before applying weightings employed by the hospital standardised mortality ratio [18, 19] to determine the Charlson score [17, 20].

The data provided was used to calculate age at admission, day of admission, season of admission, duration of hospital stay, time to procedure from admission, day of procedure and day of in-hospital death. Overall in-hospital death within 30 days was the mortality outcome of interest. The named responsible consultant for the relevant hospital spell was provided in the dataset, and consultant subspeciality was determined using information from NHS search and other subspecialist society websites [21–23]. Names were mapped to the appropriate subspeciality based on that which the consultant predominantly practiced during the year group of the study. Subspecialties were categorised as GI or other ‘general’ surgeons with the latter group consisting of breast, vascular and other non-gastrointestinal subspeciality ‘general’ surgeons working on emergency on-call rotas during the study period.

### Statistical analysis

Categorical data were summarised using frequencies and percentages, and continuous data using the mean and 95% confidence interval. Differences in case mixes between these groups were investigated using Pearson's *χ*2 test for categorical variables trends. Continuous data were assessed using the mean and 95% confidence intervals with differences between these variables determined by either students t-test or ANOVA with post-hoc testing. Comparisons of non-parametric data between two groups were performed using a Mann–Whitney U test or Kruskal–Wallis test when comparing more than 2 groups. Data were stored and processed in Excel® 2010, and analyses were undertaken using IBM SPSS Statistics v27.0 (SPSS, Chicago, Illinois, USA) and STATA 16.1 (StataCorp, College Station, Texas, USA) software. Statistical significance was defined in all cases as *P* < 0.050.

## Results

### Changes in patient characteristics over time

Over the 15 years study period, 22,137 patients were admitted with appendicitis to NHS hospitals in the North of England (Table 1). These accounted for 4.5% of acute surgical admissions. The number of presentations increased from 6844 (2002–2006) to 7507 (2012–2016, *p < *0.001). Whilst most patients were young (“Appendix 3”), the average age of presentation rose significantly (*p < *0.001). Patients were increasingly co-morbid, demonstrated by higher Charlson scores (*p < *0.001). A greater proportion were admitted via A&E latterly (39.9–60.2%, *p < *0.001) corresponding with a reduction in direct GP admissions (50.4–27.5%). An increased proportion of patients presented to smaller trusts (*p < *0.001). An increasing trend of admission under gastrointestinal surgeons was observed while admissions under other general surgery subspecialists reduced (*p < *0.001). No significant trend in admissions was evident between seasons or day of the week.

**Table 1:** Baseline characteristics, investigation and management of patients presenting with appendicitis, by study period

	Study period			Overall (*n* = 22,137)		*p*-value
	2002–2006 (*n* = 6844)	2007–2012 (*n* = 7786)	2012–2016 (*n* = 7507)			
Age	36.4 (36.0, 36.8)	36.8 (36.4, 37.2)	39.5 (39.0, 39.9)	37.6 (37.3, 37.8)		< 0.001
Sex						0.058
Male	3771 (55.1)	4192 (53.8)	3989 (53.1)	11,952 (54.0)		
Female	3072 (44.9)	3594 (46.2)	3518 (46.9)	10,184 (46.0)		
Charlson category						< 0.001
0–1	6379 (98.5)	7584 (97.4)	7204 (96.0)	21,527 (97.2)		
1–4	96 (1.4)	180 (2.3)	277 (3.7)	553 (2.5)		
≥5	9 (0.1)	22 (0.3)	26 (0.3)	57 (0.3)		
Deprivation quintile						0.169
1 (most)	1521 (26.4)	1737 (24.8)	1579 (23.9)	4887 (25.0)		
2	1319 (22.2)	1575 (22.5)	1492 (22.6)	4386 (22.4)		
3	1022 (17.2)	1222 (17.5)	1185 (18.0)	3429 (17.5)		
4	804 (13.5)	956 (13.7)	894 (13.5)	2654 (13.6)		
5 (least)	1233 (20.7)	1507 (21.5)	1450 (22.0)	4190 (21.4)		
Admission route						< 0.001
A&E	2731 (39.9)	4271 (54.9)	4280 (60.2)	11,282 (51.9)		
GP	3446 (50.4)	2903 (37.3)	1954 (27.5)	8303 (38.2)		
Consultant clinic	11 (0.2)	16 (0.2)	143 (2.0)	170 (0.8)		
Other	656 (9.6)	596 (7.7)	734 (10.3)	1986 (9.1)		
Trust size						< 0.001
Small/medium	3206 (46.8)	3967 (51.0)	4257 (56.7)	11,430 (51.6)		
Large/very large	3638 (53.2)	3819 (49.0)	3250 (43.3)	10,707 (48.4)		
Day of admission in week						0.947
Weekday	5151 (75.3)	5878 (75.5)	5662 (75.4)	16,691 (75.4)		
Weekend/bank holiday	1693 (24.7)	1,908 (24.5)	1845 (24.6)	5446 (24.6)		
Consultant subspeciality						< 0.001
Gastrointestinal surgery	2791 (40.7)	3842 (49.3)	5072 (67.5)	11,705 (52.8)		
‘Other’ general urgery	4053 (59.3)	3944 (50.7)	2435 (32.5)	10,432 (47.2)		
Season						0.198
Spring	1729 (25.3)	2055 (26.4)	1976 (26.3)	5760 (26.0)		
Summer	1825 (26.7)	2058 (26.4)	1,955 (26.0)	5838 (26.4)		
Autumn	1696 (24.8)	1934 (24.8)	1787 (23.8)	5417 (24.5)		
Winter	1594 (23.3)	1739 (22.3)	1789 (23.8)	5122 (23.1)		
Operation day						0.657
Weekday	4321 (73.2)	4895 (73.5)	4904 (74.0)	14,120 (73.6)		
Weekend/bank holiday	1578 (26.8)	1766 (26.5)	1727 (26.0)	5071 (26.4)		
Computed tomography (CT) scan performed						< 0.001
No	6787 (99.2)	6999 (89.9)	5860 (78.1)	19,646 (88.7)		
Yes	57 (0.8)	787 (10.1)	1647 (21.9)	2491 (11.3)		
Management strategy						< 0.001
Non-operative	388 (5.7)	567 (7.3)	762 (10.2)	762 (10.2)		
Appendicectomy	6398 (93.5)	7126 (91.5)	6658 (88.7)	20,182 (91.2)		< 0.001
Open	6072 (94.9)	3069 (43.1)	1015 (15.2)	10,156 (50.3)		
Laparoscopic to open	43 (0.7)	450 (6.3)	375 (5.6)	868 (4.3)		
Laparoscopic	283 (4.4)	3607 (50.6)	5268 (79.2)	9158 (45.4)		
Right hemicolectomy	58 (0.9)	93 (1.2)	87 (1.2)	238 (1.1)		< 0.001
Open	57 (98.3)	69 (74.2)	37 (42.5)	163 (68.5)		
Laparoscopic to open	0 (0.0)	17 (18.3)	32 (36.8)	49 (20.6)		
Laparoscopic	1 (1.7)	7 (7.5)	18 (20.7)	26 (10.9)		

*A&E* accident and emergency department, *GP* general practitioner. Values in parenthesis are percentages. Percentages and proportions were derived by excluding missing data from the variable. *χ*2 test for difference except ANOVA

### Changes in patient investigation and management

**Fig. 1:**
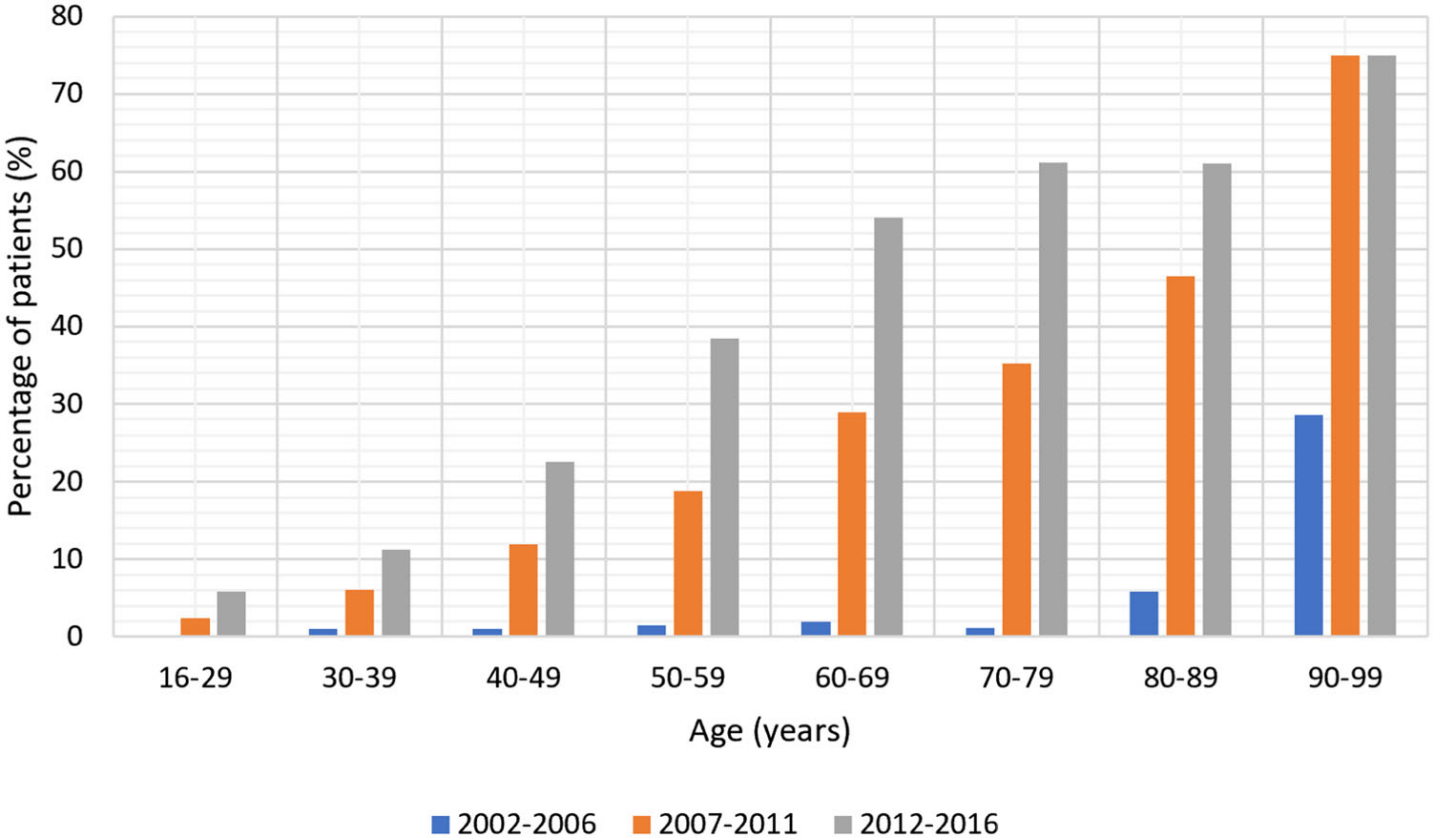
Proportion of patients with appendicitis undergoing computed tomography imaging, by age and study period

Usage of CT significantly increased over time (from 0.8 to 21.9%, *p < *0.001, Table 1). There was a rise in proportion of older patients investigated with CT (Fig. 1). It was also noted that patients from areas of greater deprivation were slightly less likely to undergo a CT scan (*p < *0.001). Patients who underwent a CT were significantly more likely to be managed non-operatively (23.8% vs. 5.7%) or undergo a right hemicolectomy (3.3% vs. 0.8%, Table 2) and spent longer in hospital (“Appendix 4”). Time to operation, amongst those who did not undergo a CT scan, remained consistently low (Table 2).

**Table 2:** Changes in the use of CT in patients presenting with appendicitis and the impact on management strategy over time, by study period

		Study period			Overall (*n* = 22,137)	*p*-value
		2002–2006 (*n* = 6844)	2007–2011 (*n* = 7786)	2012–2016 (*n* = 7507)		
CT performed (n = 2491)	Overall	57 (0.8)	787 (10.1)	1647 (21.9)	2491 (11.3)	< 0.001
	Time to procedure (days)*‡	2 (1,2)	1 (1,2)	1 (0,1)	1 (0,1)	< 0.001
	Management					< 0.001
	Non-operative management	17 (29.8)	220 (28.0)	356 (21.6)	593 (23.8)	
	Appendicectomy	37 (64.9)	534 (67.9)	356 (21.6)	1815 (72.9)	< 0.001
	Open	28 (75.7)	281 (52.6)	293 (23.6)	602 (33.2)	
	Laparoscopic to open	4 (7.0)	73 (9.3)	150 (9.1)	227 (9.1)	
	Laparoscopic	5 (8.8)	188 (23.9)	829 (50.3)	1022 (41.0)	
	Right hemicolectomy	3 (5.3)	33 (4.2)	47 (2.9)	83 (3.3)	
CT not performed (n = 19,646)	Overall	6787 (99.2)	6999 (89.9)	5860 (78.1)	19,646 (88.7)	< 0.001
	Time to procedure (days)*‡	0 (0,1)	1 (0,1)	0 (0,1)	0 (0,1)	< 0.001
	Management					< 0.001
	Non-operative management	371 (5.5)	347 (5.0)	406 (6.9)	1124 (5.7)	
	Appendicectomy	6361 (93.7)	6592 (94.2)	5414 (92.4)	18,367 (93.5)	< 0.001
	Open	6044 (95.0)	2788 (42.3)	722 (13.3)	9554 (52.0)	
	Laparoscopic to open	39 (0.6)	384 (5.8)	240 (4.4)	663 (3.6)	
	Laparoscopic	278 (4.4)	3420 (51.9)	4452 (82.2)	8150 (44.4)	
	Right hemicolectomy	55 (0.8)	60 (0.9)	40 (0.7)	155 (0.8)	

*CT* = computed tomography. Values in parenthesis are percentages unless indicated otherwise. *values displayed are median (interquartile range). Percentages and proportions were derived by excluding missing data from the variable. *χ*2 test for difference, except ‡ANOVA

**Fig. 2:**
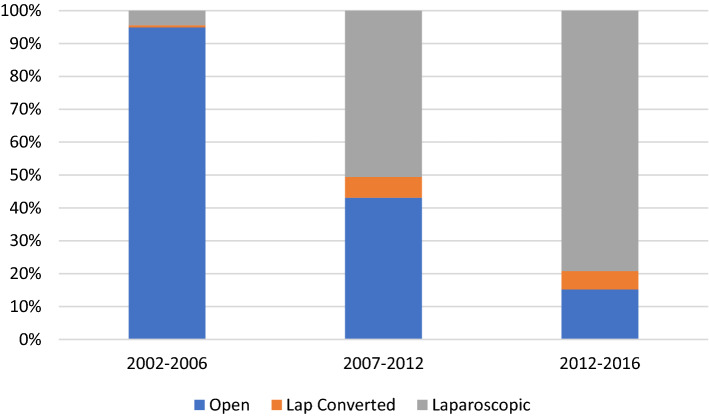
Operative approach for appendicectomy, by study period

There was a significant reduction in open operating and a rise in laparoscopic procedures (Fig. 2*, *Table 1). The proportion of laparoscopic converted to open procedures decreased from 14.0 (2002–06) to 5.8% (2012–16) (*p < *0.001). The number of patients treated non-operatively increased from 5.7 to 10.2%; they were significantly older and more co-morbid. Those who underwent a right hemicolectomy were the oldest and most co-morbid (“Appendix 5”). Patients who successfully underwent laparoscopic appendicectomy were younger, with fewer co-morbidities, admitted via A&E and under the care of a GI specialist.

### Changes in patient outcomes over time

The median length of stay in hospital significantly reduced by approximately one day (Table 3). This is reflected by the decreased length of stay in patients treated non-operatively and laparoscopically, with a slight increase in patients who were converted to open surgery from laparoscopic

**Table 3:** Clinical outcomes for patients presenting with appendicitis, by study period

	Study period			Overall (*n* = 22,137)	*p*-value
	2002–2006 (*n* = 6844)	2007–201 (*n* = 7786)	2012–2016 (*n* = 7507)		
Time to procedure (days)*‡	0 (0,1)	1 (0,1)	1 (0,1)	1 (0,1)	< 0.001
Length of stay (days) *‡	3 (2,5)	3 (2,4)	2 (2,4)	3 (2,5)	< 0.001
Non-operative	4.5 (2,8)	4 (2,7)	3 (2,6)	4 (2,7)	< 0.001
Appendicectomy	3 (2,5)	3 (2,4)	2 (2,4)	3 (2,4)	< 0.001
Laparoscopic	3 (2,4)	2 (2,4)	2 (2,3)	2 (2,3)	< 0.001
Laparoscopic to open	4 (3,5)	5 (3,7)	5 (3,8)	5 (3,7)	0.005
Open	3 (2,5)	3 (2,4)	3 (2,5)	3 (2,5)	< 0.001
Right hemicolectomy	11 (7,16)	8 (6,14)	8 (7,13)	9 (7,14)	0.249
30-day mortality	36 (0.5)	19 (0.2)	19 (0.3)	74 (0.3)	0.004
Non-operative	12 (3.1)	5 (0.9)	8 (1.1)	25 (1.5)	0.009
Appendicectomy	21 (0.3)	11 (0.2)	10 (0.2)	42 (0.2)	0.039
Laparoscopic	1 (0.4)	1 (0.0)	3 (0.1)	5 (0.1)	0.078
Laparoscopic to open	0 (0.0)	1 (0.2)	2 (0.5)	3 (0.4)	0.694
Open	20 (0.3)	9 (0.3)	5 (0.5)	34 (0.3)	0.631
Right hemicolectomy	3 (5.2)	3 (3.2)	1 (1.2)	7 (2.9)	0.365

Values in parenthesis are percentages unless indicated otherwise. *values displayed are median (interquartile range). Percentages and proportions were derived by excluding missing data from the variable. *χ*2 test for difference, except ‡ANOVA

Thirty-day mortality, though low throughout, significantly decreased over time (*p* = 0.004). Improved post-operative mortality was particularly evident amongst those treated non-operatively (2002–2006; 3.1% vs. 2012–2016; 1.1%, *p* = 0.009).

### Appendicitis in older patients

Older patients were more likely to have significant co-morbidities, undergo CT and receive non-operative management (all *p < *0.001, Table 4). Their commonest route of admission was via A&E (49.3%, *p* = 0.027). Older patients were also more likely to require a right hemicolectomy (50–59; 1.4% vs. ≥80; 4.6%, *p < *0.001).

**Table 4:** Baseline characteristics, investigation and management of older patients presenting with appendicitis, by age group

	Age range					Overall (*n* = 5469)	*p*-value
	50–59 years (*n* = 2429)		60–69 years (*n* = 1659)	70–79 years (*n* = 969)	≥80 years (*n* = 412)		
Sex							0.632
Female	1192 (49.1)	797 (48.0)		480 (49.5)	212 (51.5)	2681 (49.0)	
Male	1237 (50.9)	862 (52.0)		489 (50.5)	200 (48.5)	2788 (51.0)	
Charlson category							< 0.001
0–1	2345 (96.5)		1513 (91.2)	815 (84.1)	302 (73.3)	4975 (90.9)	
1–4	81 (3.3)		130 (7.8)	144 (14.9)	92 (22.3)	447 (8.2)	
≥5	4 (0.2)		16 (1.0)	10 (1.0)	18 (4.4)	48 (0.9)	
Deprivation quintile							0.206
1 (most)	421 (19.4)		263 (17.7)	171 (20.3)	68 (19.6)	923 (19.0)	
2	499 (23.0)		336 (22.6)	164 (19.5)	65 (18.7)	1064 (22.0)	
3	354 (16.3)		269 (18.1)	135 (16.0)	55 (15.9)	813 (16.8)	
4	358 (16.5)		225 (15.1)	152 (18.0)	57 (16.4)	792 (16.3)	
5 (least)	538 (24.8)		393 (26.4)	221 (26.2)	102 (29.4)	1254 (25.6)	
Admission route							0.027
A&E	1184 (49.8)		794 (48.7)	462 (48.6)	202 (50.0)	2642 (49.3)	
GP	955 (40.1)		661 (40.5)	365 (38.4)	142 (35.2)	2123 (39.6)	
Consultant clinic	24 (1.0)		27 (1.7)	13 (1.4)	4 (1.0)	68 (1.2)	
Other	216 (9.1)		149 (9.1)	111 (11.6)	56 (13.8)	532 (9.9)	
Trust size							0.286
Small/medium	1369 (56.3)		960 (57.9)	529 (54.6)	222 (53.9)	3080 (56.3)	
Large/very large	1061 (43.7)		699 (42.1)	440 (45.4)	190 (46.1)	2390 (43.7)	
Day of admission in week							0.383
Weekday	1810 (74.5)		1273 (76.7)	722 (74.5)	312 (75.7)	4117 (75.3)	
Weekend/bank holiday	620 (25.5)		386 (23.3)	247 (25.5)	100 (24.3)	1353 (24.7)	
Consultant subspeciality							0.981
Gastrointestinal surgery	1345 (55.4)		924 (55.7)	543 (56.0)	231 (56.1)	3043 (55.6)	
‘Other’ general surgery	1085 (44.6)		735 (44.3)	426 (44.0)	181 (43.9)	2427 (44.4)	
Season							0.661
Spring	651 (26.8)		449 (27.1)	238 (24.6)	118 (28.6)	1456 (26.6)	
Summer	654 (26.9)		436 (26.3)	264 (27.2)	105 (25.5)	1459 (26.7)	
Autumn	590 (24.3)		410 (24.7)	259 (26.7)	111 (26.9)	1370 (25.0)	
Winter	535 (22.0)		364 (21.9)	208 (21.5)	78 (18.9)	1185 (21.7)	
Operation day							0.666
Weekday	1525 (73.8)		974 (72.3)	528 (72.4)	213 (74.7)	3240 (73.2)	
Weekend/bank holiday	541 (26.2)		374 (27.7)	201 (27.6)	72 (25.3)	1188 (26.8)	
Computed tomography (CT) scan performed							< 0.001
No	1908 (78.5)		1122 (67.6)	357 (36.8)	225 (54.6)	3867 (70.7)	
Yes	522 (21.5)		537 (32.4)	357 (36.8)	187 (45.4)	1603 (29.3)	
Management strategy							< 0.001
Non-operative	241 (9.9)		230 (13.9)	216 (22.3)	131 (31.8)	818 (15.0)	
Appendicectomy	2154 (88.6)		1376 (82.9)	720 (74.3)	262 (63.6)	4512 (82.5)	
Open	1084 (50.3)		696 (50.6)	419 (58.2)	154 (58.8)	2353 (52.2)	
Laparoscopic to Open	139 (6.5)		114 (8.3)	41 (5.7)	15 (5.7)	309 (6.9)	
Laparoscopic	931 (43.2)		566 (41.1)	260 (36.1)	93 (35.5)	1850 (41.0)	
Right hemicolectomy	35 (1.4)		53 (3.2)	33 (3.4)	19 (4.6)	140 (2.6)	

*A&E* accident and emergency department, *GP* general practitioner, Values in parenthesis are percentages. Percentages and proportions were derived by excluding missing data from the variable. *χ*2 test for difference

Older patients waited longer for their operation, had a longer length of stay in hospital and a higher mortality rate after 30 days (Table 5). The time to operation almost doubled from 0.78 (50–59 years) to 1.5 (≥80 years) days (*p < *0.001) with advancing age. The oldest patients had the longest stay in hospital, averaging 12.35 days (≥80 years) compared to 4.59 days (50–59 years) (< 0.001). Those aged ≥80 also had the poorest outcomes with an 8.3% 30-day mortality rate, compared to 0.1% in younger patients (*p < *0.001).

**Table 5:** Clinical outcomes for older patients presenting with appendicitis, by age group

	Age range				Overall (*n* = 5469)	*p*-value
	50–59 years (*n* = 2429)	60–69 years (*n* = 1659)	70–79 years (*n* = 969)	≥80 years (*n* = 412)		
Days to procedure*‡	0.78 (0.72, 0.84)	1.02 (0.90, 1.14)	1.28 (1.10, 1.47)	1.50 (1.17, 1.83)	0.99 (0.93, 1.04)	< 0.001
Length of stay *$	4.59 (4.42, 4.77)	6.27 (5.85, 6.69)	7.92 (7.37, 8.47)	12.35 (10.91, 13.79)	6.27 (6.16, 6.38)	< 0.001
Non-operative	5.45 (4.84, 6.07)	8.13 (6.57, 9.68)	8.49 (7.15, 9.83)	13.46 (10.10, 16.82)	8.25 (7.43, 9.07)	< 0.001
Appendicectomy						
Laparoscopic	3.30 (3.14, 3.47)	3.96 (3.69, 4.24)	4.94 (4.43, 5.44)	9.47 (7.28, 11.64)	4.05 (3.67, 4.43)	< 0.001
Laparoscopic to open	6.24 (5.45, 7.03)	6.68 (5.83, 7.53)	9.12 (6.11, 12.13)	17.00 (6.28, 27.72)	7.35 (6.49, 8.21)	< 0.001
Open	5.28 (4.97, 5.58)	7.42 (6.67, 8.16)	9.30 (8.40, 10.20)	12.63 (10.87, 14.38)	7.15 (6.81, 7.49)	< 0.001
Right hemicolectomy	14.1 (9.3, 18.5)	14.0 (11.5, 16.4)	12.9 (9.08, 16.7)	22.3 (13.5, 31.0)	14.9 (12.8, 16.9)	0.036
30-day mortality	3 (0.1)	8 (0.5)	20 (2.1)	34 (8.3)	65 (1.2)	< 0.001
Non-operative	1 (0.4)	1 (0.4)	5 (2.3)	15 (11.5)	22 (2.7)	< 0.001
Appendicectomy	1 (0.1)	7 (0.5)	12 (1.7)	17 (6.5)	37 (0.8)	< 0.001
Laparoscopic	0 (0.0)	0 (0.0)	0 (0.0)	4 (4.3)	4 (0.2)	< 0.001
Laparoscopic to open	0 (0.0)	0 (0.0)	0 (0.0)	3 (0.17)	3 (20.0)	< 0.001
Open	1 (0.1)	7 (1.0)	12 (2.9)	10 (6.5)	30 (1.3)	< 0.001
Right hemicolectomy	1 (2.9)	0 (0.0)	3 (9.1)	2 (10.5)	6 (4.3)	0.102

Values in parenthesis are percentages. Percentages and proportions were derived by excluding missing data from the variable. *χ*2 test for difference, except ‡ANOVA or $Kruskal–Wallis

## Discussion

This study reports 22,137 cases of appendicitis across the 15 years study period in the North of England. We note significant changes in patient demographics, CT usage and operative approach.

Appendicitis is regarded as a disease of the young, and this is concordant with our findings of peak incidences between ages 16–29 (“Appendix 3”). We note an increase in the age of presentation (Table 1), similar to other studies [24, 25]. This is likely because of the increasing life expectancy over the study period [26], and with the increased usage of CT, the higher diagnosis rate of appendicitis.

A higher proportion of patients were male, which is concordant with other studies [4, 13, 14]. Males drove the increased incidence in the younger population (“Appendix 6”). The RIFT study demonstrated that two thirds of patients referred to a general surgeon with undifferentiated right iliac fossa (RIF) pain were female, yet less than 20% had a confirmed diagnosis of appendicitis [4]. It is known that gynaecological conditions in younger patients often present similarly to appendicitis and frequently lead to mislabelled differential diagnoses. Incidence of appendicitis in females is however increasing, and the rise in use of CT (Table 1) may be contributing towards improved diagnostic rates. Above 40 years, this discrepancy subsides leading to a similar, if not reversed trend thereafter. Gynaecological differential diagnoses have a lower prevalence in this age group making appendicitis a more viable diagnosis [27, 28].

We noted a significant increase in co-morbidity of patients over time (Table 1), likely associated with the rising trend of age. The increase in admission under a GI subspecialist reflects the changes in surgical training and a move towards more GI sub-specialists working a greater proportion of emergency cover [29].

Proposed international guidelines [8, 9] set out a diagnostic pathway for appendicitis. Patients can be categorised into low, intermediate, or high risk based on clinical biochemistry and risk scores using the Alvarado Score (AS) [30], Appendicitis Inflammatory Response Score (AIRS) [31] or Adult Appendicitis Score (AAS) [32]. Recommendations are to use AAS in women and AIRS in men [4]. Alone, these scores provide limited information, so imaging is recommended in higher risk patients to clarify diagnostic uncertainty [8, 9].

CT is widely used due to greater availability, reduced cost and high sensitivity and specificity [6]. All hospitals in the North of England gained access to out-of-hours radiology services during the study period, where CT reporting may have aided diagnosis and informed decision making. It could be suggested that CT can identify patients with uncomplicated appendicitis who may benefit from non-operative management [6, 8]. Our results suggest that the rise in CT usage was predominantly driven by older patients (Fig. 1). Guidelines recommend CT to rule out alternative diagnoses such as malignancy which can mask the presentation of appendicitis [33].

CT has a high specificity but low sensitivity for differentiating complicated from uncomplicated appendicitis lowering accuracy for identifying patients who can be medically managed [34]. Guidelines suggest surgical management should be pursued in all cases unless contraindicated [6, 8, 9]. We propose that CT is useful for guiding surgical management rather than identifying patients with uncomplicated disease who could be managed non-operatively, as previously suggested [6].

The increase in laparoscopy and reduction in open surgery is concordant with the current gold standard treatment [6, 8, 9]. We identified increasing success in laparoscopic approach, with a significant reduction in laparoscopic converted to open appendicectomies (14.0–5.8%, *p < *0.001). One meta-analysis found similar results; conversion rates between 20–45% before 2000 and 0–18% over the last decade [35]. The success of laparoscopy can be attributed in part to the increase in GI subspecialists treating patients who have likely found confidence with laparoscopy from their elective practice.

Non-operative management of appendicitis became increasingly relevant in the current SARS-Cov-2 pandemic. The proportion of patients managed non-operatively rose to 64% during the peak of the pandemic [36]. COVID-19 causes an inflammatory viral-induced cytokine storm increasing surgical mortality risk [37]. Despite little evidence supporting viral transmission through aerosolised procedures, studies recommend modifications to standard practice to minimise risk of transmission [38]. Benefits of antibiotic therapy as an alternative to surgery have been explored [36, 39]. Recent literature found that although antibiotics had a lower infection rate, their rate of reoperation and disease recurrence was higher than surgical intervention [14, 40]. Poorer quality of life has been discovered with antibiotics alone; no difference has been identified in hospital length of stay [41]. Consequently, laparoscopic surgery remains the gold standard treatment [6, 8, 9] as supported by our results (Table 3).

The rise in patients managed non-operatively may be due to the changing demographics of the cohort over time. Our results demonstrated that patients receiving non-operative management were amongst the oldest and most comorbid (“Appendix 5”), and these factors will commonly lead to an increased risk from surgical intervention. One meta-analysis described a reduced complication rate (OR 0.21–0.51) with conservative management compared to any operative approach in elderly patients, emphasising the potential age-related risk of surgical intervention [40]. Public preference for antibiotic treatment to avoid an operation is also notable from contemporary literature [42] and a shift from paternalistic medicine, towards patient-centred care may also be contributing to the rising non-operative treatment.

The improved length of stay is likely driven by the rising popularity of laparoscopy and reduction in overall mortality [8]. Poorest outcomes were among those managed with a right hemicolectomy or non-operatively however, these patients were more elderly and comorbid (“Appendix 5”). Length of stay and overall mortality improved across the study period in both age groups.

Appendicitis is complicated in the elderly. Differential diagnoses of RIF pain increase with age as often presentation can be vague. Previous literature indicates that CT is preferred to investigate as it improves diagnostic accuracy and reduces negative appendicectomy rates [43]. When surgery is chosen, it incurs longer operation waiting times and length of stay in hospital, which may reflect reluctance to operate or diagnostic challenges [43], reinforced by our findings.

The hospital episode statistics data utilised, relies on accurate clinical coding, which depends on good documentation. In large datasets like this, small individual inaccuracies in coding are likely to be insignificant and unlikely to bias overall results. These data don’t allow accurate analysis of negative appendicectomy rates as histological data was unavailable, nor were details of complications, readmissions, or antibiotic use in this population. Data on clinical biochemistry and calculated risk scores for individual patients would have allowed risk stratification using AAS, AIRS and AS but unfortunately this was not able to be performed for this study. Details of a broader population with undifferentiated RIF pain would have allowed us to comment on the proportions of patients who went on to be diagnosed with appendicitis. Admissions are coded by the consultant responsible for care, rather than the operative consultant. The majority of appendicectomies in public hospitals are performed by non-consultant grade surgeons [44], thus the two may differ limiting conclusions drawn on operative influence.

Our results highlight improvements made in managing appendicitis over time. These results are highly suggestive that improved outcomes are due to laparoscopic operating and CT usage, emphasising the importance of laparoscopic appendicectomy as the gold standard treatment for appendicitis [6, 8, 9].

## Appendix 1: A map of the Northeast of England and its relevant trusts

Source: Calderdale and Kirklees 999 Call for the NHS, 2019 [45].

## Appendix 2: Data fields requested from the National Health Service Foundation Trusts’ Caldicott Guardian

**Table:**

Demographic fields	Age, sex, postcode
Co-morbidity fields	ICD-10 diagnosis 2 onwards
Diagnosis/operation fields	Primary diagnosis (ICD-10 diagnosis 1), operation date, operation type (OPCS codes for operation 1 onwards), consultant name
Outcome fields	Admission date and source, discharge date and location, mortality (time to death)

## Appendix 4: Outcomes for patients presenting with appendicitis by CT utilisation

**Table:**

	CT performed (*n* = 2491)	CT Not performed (*n* = 19,646)	Overall (*n* = 22,137)	*p* value
Length of stay**‡*	5 (3,8)	3 (2,4)	3 (2,5)	< 0.001
Non-operative	5 (3,7)	3 (2,6)	4 (2,7)	< 0.001
Appendicectomy	5 (3,8)	3 (2,4)	3 (2,4)	< 0.001
Laparoscopic	4 (2,6)	2 (2,3)	2 (2,3)	< 0.001
Laparoscopic to open	7 (5,10)	4 (3,6)	5 (3,7)	< 0.001
Open	6 (4,10)	3 (2,4)	3 (2,5)	< 0.001
Right Hemicolectomy	10 (8,14)	9 (6,14)	9 (7,14)	0.144
30-day mortality	17 (0.7)	57 (0.3)	74 (0.3)	0.001
Non-operative	9 (1.5)	16 (1.4)	25 (1.5)	0.877
Appendicectomy	7 (0.4)	35 (0.2)	42 (0.2)	0.082
Laparoscopic	2 (0.2)	3 (0.0)	5 (0.1)	0.038
Laparoscopic to open	1 (0.5)	2 (0.3)	3 (0.4)	0.691
Open	4 (0.7)	30 (0.3)	34 (0.3)	0.149
Right Hemicolectomy	1 (1.2)	6 (3.9)	7 (2.9)	0.246

Values in parentheses are percentages unless indicated otherwise; *values are median (interquartile range). Percentages and proportions were derived by excluding missing data from the variable. *χ*2 test for difference, except ^‡^Kruskal–Wallis.

## Appendix 5: Characteristics of patients presenting with appendicitis, by management approach

**Table:**

Management approach							
	Non-Operative (*n* = 1717)	Appendicectomy (*n* = 20,182)			Right Hemicolectomy (*n* = 238)	Overall (*n* = 22,137)	*p*-value
		Open (*n* = 10,155)	Laparoscopic to Open (*n* = 868)	Laparoscopic (*n* = 9158)			
Age*‡	48 (3166)	33 (2248)	43 (2756)	31 (2246)	56.5 (4069)	34 (2249)	< 0.001
Sex							0.349
Female	804 (46.8)	4202 (41.4)	416 (47.9)	4643 (50.7)	119 (50.0)	10,184 (46.0)	
Male	913 (53.2)	5953 (58.6)	452 (52.1)	4515 (49.3)	119 (50.0)	11,952 (54.0)	
Charlson Category Score							< 0.001
0–1	1574 (91.7)	9944 (97.9)	832 (95.9)	8992 (98.2)	185 (77.7)	21,527 (97.2)	
2–4	125 (7.3)	197 (1.9)	34 (3.9)	159 (1.7)	38 (16.0)	553 (2.5)	
≥5	18 (1.0)	15 (0.2)	2 (0.2)	7 (0.1)	15 (6.3)	57 (0.3)	
Season							0.613
Spring	470 (27.4)	2624 (25.8)	216 (24.9)	2395 (26.2)	55 (23.1)	5760 (26.0)	
Summer	464 (27.0)	2599 (25.6)	257 (29.6)	2456 (26.8)	62 (26.1)	5838 (26.4)	
Autumn	405 (23.6)	2388 (23.5)	200 (23.0)	2363 (25.8)	61 (25.6)	5417 (24.5)	
Winter	378 (22.0)	2545 (25.1)	195 (22.5)	1944 (21.2)	60 (25.2)	5122 (23.1)	
Deprivation Quintile							0.275
1 (most)	328 (22.3)	2321 (26.1)	188 (24.3)	2004 (24.3)	46 (23.0)	4887 (25.0)	
2	351 (24.0)	1993 (22.5)	172 (22.3)	1828 (22.2)	42 (21.0)	4386 (22.4)	
3	264 (18.0)	1514 (17.1)	141 (18.3)	1479 (18.0)	31 (15.5)	3429 (17.6)	
4	196 (13.4)	1215 (13.7)	105 (13.6)	1109 (13.5)	29 (14.5)	2654 (13.6)	
5 (least)	326 (22.3)	1834 (20.6)	166 (21.5)	1812 (22.0)	52 (26.0)	4190 (21.4)	
Admission route							< 0.001
A&E	800 (47.5)	4439 (43.9)	478 (55.4)	5467 (61.8)	98 (41.5)	11,282 (51.9)	
GP	621 (36.8)	4658 (46.1)	331 (38.4)	2594 (29.3)	99 (41.9)	8303 (38.2)	
Consultant Clinic	37 (2.2)	22 (0.2)	2 (0.2)	104 (1.2)	5 (2.1)	170 (0.8)	
Other	228 (13.5)	987 (9.8)	52 (6.0)	685 (7.7)	34 (14.5)	1986 (9.1)	
Consultant subspeciality							0.064
General Surgeon	786 (45.8)	5919 (58.3)	431 (49.7)	3168 (34.6)	128 (53.8)	10,432 (47.1)	
GI Sub-specialist	931 (54.2)	4237 (41.7)	437 (50.3)	5990 (65.4)	110 (46.2)	11,705 (52.9)	
Trust Size							< 0.001
Small/Medium	998 (58.1)	4979 (49.0)	435 (50.1)	4890 (53.4)	128 (53.8)	11,430 (51.6)	
Large/Very Large	719 (41.9)	5177 (51.0)	433 (49.9)	4268 (46.6)	110 (46.2)	10,707 (48.4)	
Day of week of admission							< 0.001
Weekday	1356 (79.0)	7616 (75.0)	626 (72.1)	6891 (75.2)	202 (84.9)	16,691 (75.4)	
Weekend or Bank Holiday	361 (21.0)	2540 (25.0)	242 (27.9)	2267 (24.8)	36 (15.1)	5446 (24.6)	
Day of week of operation							0.044
Weekday	116 (82.3)	6907 (73.5)	537 (72.0)	6392 (73.6)	168 (76.0)	14,120 (73.6)	
Weekend of Bank Holiday	25 (17.7)	2486 (26.5)	209 (28.0)	2298 (26.4)	53 (24.0)	5071 (26.4)	
Computed Tomography scan performed							< 0.001
No	1124 (65.5)	9554 (94.1)	663 (76.4)	8,150 (89.0)	155 (65.1)	19,646 (88.7)	
Yes	593 (34.5)	602 (5.9)	205 (23.6)	1008 (11.0)	83 (34.9)	2491 (11.3)	

Values in parentheses are percentages unless indicated otherwise; *values are median (interquartile range). Percentages and proportions were derived by excluding missing data from the variable. A&E, accident and emergency department; GP, general practitioner; GI, gastrointestinal. *χ*2 test for difference, except ‡Kruskal–Wallis*.*
